# Islands in the ice: Potential impacts of habitat transformation on Antarctic biodiversity

**DOI:** 10.1111/gcb.16331

**Published:** 2022-07-24

**Authors:** Jasmine R. Lee, Melinda J. Waterman, Justine D. Shaw, Dana M. Bergstrom, Heather J. Lynch, Diana H. Wall, Sharon A. Robinson

**Affiliations:** ^1^ British Antarctic Survey NERC Cambridge UK; ^2^ Securing Antarctica's Environmental Future, School of Biology and Environmental Science Queensland University of Technology Brisbane QLD Australia; ^3^ Securing Antarctica's Environmental Future, School of Earth, Atmospheric and Life Sciences University of Wollongong Wollongong New South Wales Australia; ^4^ Australian Antarctic Division, Department of Agriculture Water and the Environment Kingston TAS Australia; ^5^ Global Challenges Program University of Wollongong Wollongong New South Wales Australia; ^6^ Department of Ecology and Evolution Stony Brook University Stony Brook New York USA; ^7^ Department of Biology and School of Global Environmental Sustainability Colorado State University Fort Collins Colorado USA

**Keywords:** Antarctica, biodiversity, biotic homogenization, climate change, connectivity, ice‐free, non‐native species

## Abstract

Antarctic biodiversity faces an unknown future with a changing climate. Most terrestrial biota is restricted to limited patches of ice‐free land in a sea of ice, where they are adapted to the continent's extreme cold and wind and exploit microhabitats of suitable conditions. As temperatures rise, ice‐free areas are predicted to expand, more rapidly in some areas than others. There is high uncertainty as to how species' distributions, physiology, abundance, and survivorship will be affected as their habitats transform. Here we use current knowledge to propose hypotheses that ice‐free area expansion (i) will increase habitat availability, though the quality of habitat will vary; (ii) will increase structural connectivity, although not necessarily increase opportunities for species establishment; (iii) combined with milder climates will increase likelihood of non‐native species establishment, but may also lengthen activity windows for all species; and (iv) will benefit some species and not others, possibly resulting in increased homogeneity of biodiversity. We anticipate considerable spatial, temporal, and taxonomic variation in species responses, and a heightened need for interdisciplinary research to understand the factors associated with ecosystem resilience under future scenarios. Such research will help identify at‐risk species or vulnerable localities and is crucial for informing environmental management and policymaking into the future.

## Graphical abstract

Ice‐free habitat in Antarctica is predicted to expand as temperatures warm and ice melts. It is uncertain how terrestrial biodiversity will respond to the habitat changes. Here we use current knowledge to propose four hypotheses on how species might respond to climate‐induced habitat change.

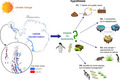

Climate change is driving global species distributional shifts, loss of diversity, dramatic habitat changes, and ecosystem collapse (Bergstrom et al., 2021; Pecl et al., 2017). Antarctica is no exception. Despite only covering <1% of the continent (Brooks et al., 2019; Burton‐Johnson et al., 2016), permanently ice‐free land provides crucial habitat for most of Antarctica's terrestrial biodiversity, including its iconic seabirds (Convey et al., 2014). Ice‐free areas occur as coastal oases, cliffs, or nunataks, and often form small patches or islands of rock and soil (habitat islands; Frenot et al., 2005) in a matrix of ice or snow. Under moderate to severe climate forcing scenarios (RCP4.5; 8.5), ice‐free areas are predicted to drastically expand in some parts of the continent—namely the Antarctic Peninsula—by the end of the century (Lee et al., 2017). This will result in an increase in available habitat and increasing connectivity between habitat patches (Lee et al., 2017), with potentially profound impacts on biodiversity. While we have many hypotheses on potential impacts, we lack sufficient quantitative evidence to test them. Here we outline some of these hypotheses (Figure 1) and highlight areas where further research is needed to understand the impacts of habitat expansion.

**FIGURE 1:**
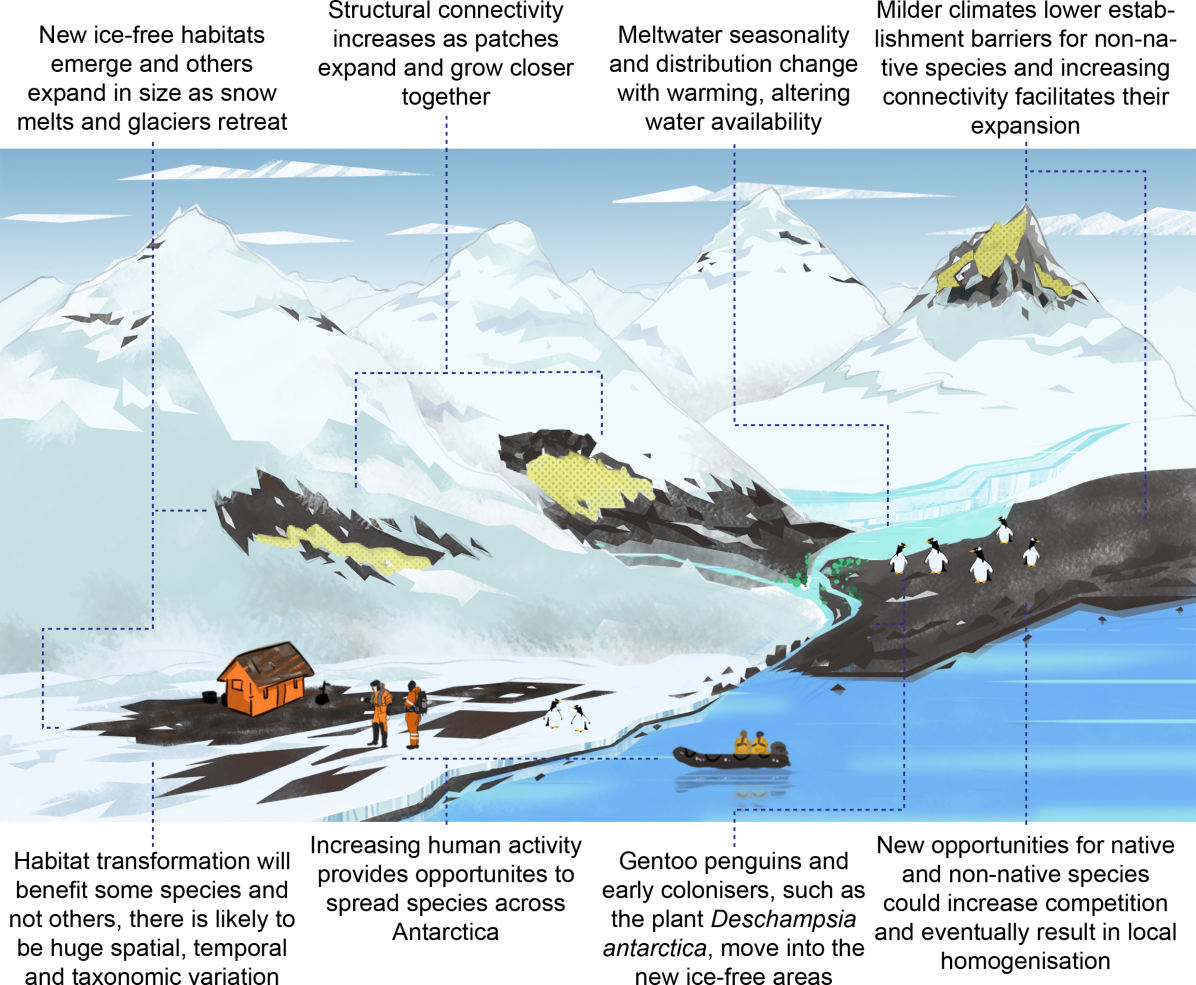
Potential biodiversity impacts of expanding ice‐free habitat in terrestrial Antarctica as discussed in this paper. Yellow frost indicates places where we hypothesize that retreating snow and ice could lead to drying ice‐free areas if water availability changes and meltwater drainage from above decreases.

## 1 INCREASING HABITAT AVAILABILITY CREATES NEW AND NOVEL SPACE FOR BIODIVERSITY EXPANSION, YET NOT ALL PATCHES WILL BE CREATED EQUAL

Thousands of square kilometres of new ice‐free area will create new habitats ripe for colonization. However, not all new patches will be equally suitable for Antarctic biodiversity. Abiotic factors are key determinants of the diversity and distribution of Antarctic terrestrial species, with water availability and soil properties dominant drivers (Adams et al., 2014; Convey, 1996; Convey et al., 2014; Dragone et al., 2021; Kennedy, 1993; Wasley et al., 2012).

Current water sources predominantly come from fresh fallen or blown snow (or rain), or past accumulation stored in snow and ice banks, as well as local glaciers. Essential meltwater can be delivered either as a steady stream or as a pulsed event to vegetation, microbial, invertebrate, and limnetic communities, and it drives clear spatial structure in biodiversity (Gooseff et al., 2003, 2017; Malenovský et al., 2017; Robinson et al., 2018, 2020). For example, in the moss communities of East Antarctica, more hydric species, such as *Schistidium antarctici*, thrive in the path of the seasonal melt stream, whereas species with higher desiccation tolerance and submergence intolerance, such as *Ceratodon purpureus*, flourish at higher elevations (Robinson et al., 2018; Wasley et al., 2012). Over the last two decades, increased westerly winds have led to increased drying and reduced inundation, with subsequent reduction of hydric species and increase in desiccation tolerant species (Robinson et al., 2018). Moss cores from the Antarctic Peninsula also indicate a general drying trend in the decades leading up to 2000 (Amesbury et al., 2017).

Potential increases in ice‐free area may drive corresponding changes in meltwater distribution and abundance. Meltwater often occurs on the periphery of patches, where snow and ice are present, and where the reduced albedo effect drives further melting of adjacent snow (Gooseff et al., 2003; Kingslake et al., 2017). As patches expand in size, moisture gradients across patches will be exacerbated, where the middle of an expanding patch may dry out as meltwater sources shift further away. This will drive subsequent changes in community distributions, such as vegetation migration towards habitat edges. These effects are spatially variable—there is already a stark contrast in climates between the Peninsula, which is wetter and warmer, and continental Antarctica, which is colder and drier (Kennedy, 1993). The increased water availability in the Peninsula may buffer edge effects as ice‐free patches grow in size, and the increasing frequency of liquid precipitation (Kirchgäßner, 2011; Robinson et al., 2020) may further alter and offset edge effects if rain is sufficient to sustain seasonal plant growth windows (Convey, 2006). However, rain falling on ice may run off into the ocean and remain unavailable to the terrestrial ecosystem (Kirchgäßner, 2011). Furthermore, changes in the seasonal distribution of water may create feast‐famine type availability—localized floods in spring or early summer due to warmer temperatures, followed by periods of drought once snowbank supplies are exhausted (Convey, 2003, 2006; Robinson et al., 2020). Early onset of spring snow melt, with higher peak flows at the expense of summer flows, is identified as a threat in other snow‐dominated regions globally (IPCC, 2021).

Climate change is leading to an increased frequency of extreme events. An anomalous flood year in the McMurdo Dry Valleys, currently Antarctica's largest ice‐free area, following a cooling period produced the greatest amount of glacial meltwater in over three decades (Gooseff et al., 2017). The response of terrestrial and freshwater communities was highly variable. Cyanobacterial mats dominated by *Nostoc* spp. quickly increased production, while mats of *Phormidium* spp. slowly reduced in abundance before gradually recovering from the flood stress over subsequent years (Gooseff et al., 2017). Shifts in soil nematode populations also lagged by several years in response to the increased water availability (Gooseff et al., 2017). The interactions between ice‐free area expansion and changes to water availability clearly play a major role in driving community composition and distribution, and research targeted towards these connections will be crucial for predicting climate change impacts on Antarctic ecosystems. Furthermore, long‐term observing systems such as the existing long term ecological research (LTER) system in the Dry Valleys (see Gooseff et al., 2003, 2017) and the proposed Antarctic Nearshore and Terrestrial Observing System (https://www.scar.org/science/antos/home/) should plan to either relocate monitored sites and or expand observed sites to accommodate such future changes.

Other abiotic factors influencing the ecological functionality of new ice‐free habitats are the properties of newly exposed soils, including pH, salinity, and levels of organic matter and nutrients, which will determine initial habitat suitability for vegetation, microfauna, and microbes (Barrett et al., 2006; Courtright et al., 2001; Dragone et al., 2022; Smykla et al., 2018). Multiple environmental and geographical factors determine these soil properties, including elevation, temperature, age of the soil, distance to coast, distance to the nearest vertebrate colony, and geological‐, glacial‐, and sea‐level history (Adamson & Pickard, 1986; Diaz et al., 2021; Dragone et al., 2022; Franco et al., 2021; Smykla et al., 2007). Because many Antarctic soils are nutrient poor, breeding seabird colonies have a large influence on terrestrial communities around coastal Antarctica, where nitrogen‐rich guano provides essential nutrients for biodiversity (Bokhorst et al., 2019; Casanovas et al., 2015; Smykla et al., 2007, 2015). Being very close to a large colony is undesirable for most species, however, there are exceptions with some species thriving in these environments, for example, the alga *Prasiola crispa* and nematode *Panagrolaimus davidii*, as well as many microbes (Barrett et al., 2006; Broady, 1989; Smykla et al., 2007, 2015). Nutrients are dispersed far beyond the colonies' immediate vicinity via wind and water (Bokhorst et al., 2019; Erskine et al., 1998; Smykla et al., 2007). Abandoned penguin colonies, some thousands of years old, also provide crucial sources of nitrogen for colonizing plants (Tatur et al., 1997; Wasley et al., 2012).

Given their obvious importance, a crucial question is whether soils of newly exposed ice‐free areas will be suitable for some terrestrial communities, for example, communities including mosses and vascular plants, unless they are first colonized by seabirds or seals. Current studies indicate this is not likely to be a constraint in some regions for at least two reasons.

First, some penguin species rapidly and opportunistically colonize newly deglaciated areas for breeding. King penguins at St. Andrews Bay, South Georgia have swiftly expanded their population and breeding ground into newly exposed ice‐free areas following glacial retreat (Foley et al., 2018), as have gentoo penguins breeding on the Western Antarctic Peninsula (Herman et al., 2020), and Adélie penguins on Beaufort Island in the Ross Sea (Larue et al., 2013). The movement of penguins into new ice‐free areas may then facilitate the establishment of other species in these areas via nutrient deposition, or perhaps, allow their old breeding sites to be colonized upon abandonment (Casanovas et al., 2013; Smykla et al., 2007; Tatur et al., 1997).

Second, in inland ice‐free areas, it is generally the older soils that support the least biodiversity (Diaz et al., 2021; Franco et al., 2021; Lyons et al., 2016). Multiple studies from the Shackleton and Beardmore Glaciers in Victoria Land found that soils exposed before the Last Glacial Maximum were generally inhospitable to soil invertebrates, sometimes even microbes, largely due to an accumulation of atmospherically deposited nitrate salts (Dragone et al., 2021; Franco et al., 2021; Lyons et al., 2016). Indeed, the sites with the highest abundance and richness of invertebrates were on the soils exposed by recent glacial recession (Franco et al., 2021). These studies indicate that newly exposed soils, with decreased soil salinity and increased soil moisture, may be preferable for some terrestrial biodiversity. In contrast, the Windmill Islands' lush moss beds are found on weathered, ancient penguin colonies. Here, newly exposed soils may become poorer in nutrients but closer to new water sources, while current sites rich in nutrients may in future lack sufficient water (Wasley et al., 2012).

Determining habitat suitability of newly exposed soils is important for understanding the potential redistribution of biodiversity with habitat change. Are patches that are uncovered or undergo expansion via snow or ice melt equivalent to those with glacial recession? Are new inland ice‐free patches, with little to no marine nutrient input, equivalent to those exposed on the coast? Are newer soils or ‘young patches’ in the maritime Antarctic and East Antarctic equally suitable as they appear to be in Victoria Land? Are they suitable for all taxa, or only some? What role do seabirds and marine mammals play as drivers of community ecology in and on newly established soils, and what factors influence the likelihood of colonization by seabirds?

## 2 ICE‐FREE AREA EXPANSION LEADS TO INCREASING STRUCTURAL CONNECTIVITY, ALTHOUGH NOT NECESSARILY INCREASING OPPORTUNITY FOR ESTABLISHMENT

Antarctica's ice‐free areas are often isolated islands in a sea of ice and snow (Frenot et al., 2005; Lee et al., 2017). As ice‐free areas expand in size and new patches emerge, the distance between patches will decrease, thus increasing connectivity (Lee et al., 2017). However, whether an increase in structural connectivity (physical connectivity of a landscape) leads to an increase in functional connectivity (the response of the species to the landscape dependent on intrinsic traits and behaviour) is likely to be highly variable amongst taxa (Auffret et al., 2015; Bergstrom et al., 2006).

Ice melt and glacial recession will reduce some, though not all, structural barriers to dispersal. Although ice and snow form some of the largest physical barriers on the continent (Beet et al., 2016; Collins et al., 2019; Stevens & Hogg, 2003), mountain ranges also present challenges for dispersal (Biersma et al., 2018; Colesie et al., 2014). While seabirds can sometimes traverse across vast distances of ice and snow, plants, microbes, and invertebrates generally rely on wind and water as passive dispersal vectors, as well as on assisted dispersal via animals and humans (Convey, 2017; Hughes et al., 2006, 2019; Nkem et al., 2006). While the mechanisms of dispersal are reasonably well understood, direct measurements of dispersal distances are challenging, though some minimal distances can be inferred. Studies from fellfield environments on Signy Island, South Orkney Islands, examined dispersal potential and the spore/propagule sizes of lichen, mosses and algae (Marshall, 1996; Marshall & Chalmers, 1997; Marshall & Convey, 1997). Distances were not examined directly, though lichen vegetative propagules were commonly observed in the air up to ~50 m from the closest possible source (Marshall, 1996) and snow algae were trapped 16 km from their main source (Marshall & Chalmers, 1997). Studies on the collembola *Cryptopygus antarcticus* have found it can survive short‐distance wind dispersal events (up to ~8 km) between ice‐free patches (Hawes et al., 2007; Nkem et al., 2006), while water rafting studies suggest it has the potential for long‐distance dispersal (Hawes et al., 2008). A Dry Valleys study confirmed that desiccated nematodes are successfully dispersed via wind (Nkem et al., 2006).

Genetic data also provide information on dispersal distances, though it is hard to determine exact distances or time scales. Genetic studies of the two vascular plants, *Colobanthus quitensis* and *Deschampsia antarctica*, suggest these angiosperms dispersed from southern South America (possibly from or via South Georgia for *C. quitensis*) in the mid‐late Pleistocene (Biersma et al., 2020; Fasanella et al., 2017). Studies of temperate mosses and cosmopolitan thermophilic microbes found at geothermal sites provide further support that long‐distance wind facilitated dispersal (up to thousands of km) over ice and ocean is possible (Herbold et al., 2014; Muñoz et al., 2004; Skotnicki et al., 2000, 2001). However, the emerging pattern suggests many Antarctic moss species were dispersed in past interglacials; thus, long‐distance dispersal appears to be a rare event (Biersma et al., 2017, 2018). This appears to be true for some microbes as well as mosses, where studies on fungi and bacteria in the McMurdo Dry Valleys indicate that dispersal events from continents outside of Antarctica are rare (Archer et al., 2019). Furthermore, the substantial spatial structure of communities within the region indicates that there are also local dispersal limitations for many organisms (including diatoms and fungi), although bacteria appear less limited in their dispersal potential (Archer et al., 2019; Sakaeva et al., 2016). For invertebrates, genetic sequencing of Victoria Land springtails revealed that ice also forms substantial geographic dispersal barriers, with some populations being highly diverged across even relatively small distances (e.g. the Tucker Glacier of <16 km across; Collins et al., 2019). In contrast, Adams et al. (2007) found no genetic differences between populations of the nematode *Scottnema lindsayae* over 700 km apart, separated by multiple glaciers and 5° in latitude, implying high dispersal potential, possibly facilitated by katabatic winds.

Few studies demonstrate evidence of animal or human‐assisted dispersal of native species over short or long distances within Antarctica, though this is a difficult phenomenon to document and the absence of evidence is not evidence of absence. For example, birds (e.g. skuas, sheathbills and gulls) are known to transport propagules of native plants for nesting materials across relatively short distances (Parnikoza et al., 2018; Quintana et al., 2001), and genetic analysis of the collembola *Gomphiocephalus hodgsoni* yielded evidence that its long‐distance dispersal across McMurdo Sound was likely assisted by birds or humans (Stevens & Hogg, 2003). However, there are numerous reports of non‐native organisms arriving from outside the continent with human facilitated transport (e.g. Hughes et al., 2010, 2013; Huiskes et al., 2014; Lee & Chown, 2011), some of which even survived transport and were later eradicated (Bergstrom, Sharman, et al., 2018; Pertierra, Aragón, et al., 2017).

These studies clearly indicate that many terrestrial species have great potential to undertake short and long‐distance dispersal within the continent (sometimes crossing major structural barriers). Equally clear is that these abilities vary between taxa; for vegetative propagules and spores, particle size appears to have varying effects on dispersal (Marshall, 1996; Marshall & Convey, 1997), while desiccation tolerance among invertebrates appears linked to greater dispersal potential and reduced risk of mortality enroute (Hawes et al., 2007; Nkem et al., 2006). Consequently, changes in structural connectivity do not appear to change functional connectivity for all taxa, though further research in this space is needed.

While many species have the potential to reach newly ice‐free areas based on dispersal ability, this is only one aspect of colonization. Whether a species establishes in a new location will depend on the intrinsic traits of the species and on the suitability of the habitat which, as discussed above, is likely to vary between patches (Bergstrom et al., 2006; Hughes et al., 2006, 2019; Virginia & Wall, 1999). So, what is likely to colonize these new ice‐free areas, and is it always the same species? Antarctic scientists realized decades ago that recently deglaciated ice‐free areas formed a perfect in situ experiment for assessing colonization processes, though studies tended to focus on a specific taxa or group (e.g. Gryziak, 2009; Lindsay, 1973; Valladares & Sancho, 1995), particularly vegetation. Studies at the ecosystem level and comparison across regions or environments to identify universal patterns are still lacking.

Long‐term work has demonstrated how quickly vegetative communities can change in response to glacial retreat (Smith, 1984; Frenot et al., 1995; Olech, 2010; Olech & Massalski, 2001; Olech et al., 2011). Pioneer colonizers in the fellfields of retreating glaciers included the mosses *Bryum pseudotriquetrum*, *B. argenteum* and *C. purpureus*, alongside the two vascular plants *D. antarctica* and *C. quitensis*. Lichens generally follow in subsequent stages of succession (Olech, 2010; Olech et al., 2011; Smith, 1984), though *Usnea antarctica* has been noted rapidly colonizing boulders (Lindsay, 1971). In contrast, some water‐loving species declined over time or vanished completely, in particular the mosses *Warnstorfia sarmentosa* and *Brachythecium austrosalebrosum*, several lichens including *Leptogium puberulum* and *Polyblastia gothica*, and ascomycota fungi *Octospora arctowski*. These communities generally require permanent water sources and with glacial retreat and disappearance of seasonal snow patches the meltwater supply dried up, leaving them to be overtaken by monocultures of *D. antarctica* (Olech, 2010). Lindsay (1978) suggested the initial time from lichen colonization to a complex community would take 200 years or more, and Favero‐Longo et al. (2012) observed how vegetation communities mature in soils of varying ages post‐deglaciation. As discussed above, some penguin species are very capable of colonizing new ice‐free areas, though less information appears to be available on microbial and invertebrate colonization and succession processes. Mite distribution appears closely linked to that of plants, with prostigmatid mites observed to be primary colonists and Oribatid mites appearing later at least 30 years post‐glacial retreat (Gryziak, 2009). Another example, at Hurd Glacier, on Livingston Island, found community composition of microbial colonizers differed based on substrate (rock or soil; Garrido‐Benavent et al., 2020). There is also still much to be understood regarding the stages of colonization and community succession, including the role that nurse plants and soil colonizers play in community establishment, and their interplay with environmental drivers (Combrinck et al., 2020; Klanderud, 2005; Le Roux et al., 2013). Currently, ice‐free vegetation is dominated by lichen assemblages, but if these are slow to establish, then other faster species may come to dominate communities. Understanding colonization processes and how these might be impacted by global change is one of the keys for predicting the future community compositions of ice‐free areas.

We anticipate that the expansion of ice‐free areas will expose more habitat for spores, propagules and desiccated invertebrates to settle on, and that these changes will occur alongside a decrease in distance between patches. Together, these factors should increase the rate of successful dispersal events. Alongside habitat expansion, changes in dispersal vectors are also expected. Increasing meltwater is likely to further facilitate local dispersal of terrestrial species (Hawes, 2011; Skotnicki et al., 1999) and changing winds may help or hinder dispersal depending on direction (with upwind sites less accessible; Marshall & Chalmers, 1997; Vega et al., 2019). Furthermore, changing wind patterns across the Southern Hemisphere (Robinson & Erickson, 2015) have been shown to move more dust into Antarctic locations (Cataldo et al., 2013; McConnell et al., 2007). This dust demonstrates the potential for propagules to reach Antarctica or increase connectivity between existing ice‐free areas and could introduce additional nutrients into the environment (Diaz et al., 2018; Šabacká et al., 2012). Increasing opportunities for human‐facilitated dispersal are a cause of major concern as human activities grow and the Antarctic climate warms (Barnes et al., 2006; Bergstrom, 2022; Hughes et al., 2006, 2019; Vega et al., 2019). This begs the question: is more connectivity good or bad for Antarctica's native species? The somewhat unsatisfactory answer is that it depends on the nature of the species and the location within Antarctica. Some native species are better dispersers and colonizers than others, as such they may have more opportunity to expand their ranges with increasing habitat and structural connectivity. However, this changing landscape may also facilitate negative interactions if non‐native species expand, or remote populations become more accessible (see below).

## 3 MILDER CLIMATES COMBINE SYNERGISTICALLY WITH NEW HABITAT TO CREATE OPPORTUNITIES FOR NON‐NATIVE SPECIES ESTABLISHMENT, BUT MAY ALSO LENGTHEN ACTIVITY WINDOWS FOR ALL SPECIES

Not only will there be new ice‐free habitat available, but global change will also directly influence Antarctic climate with predicted increases in temperature and precipitation (Lee et al., 2017; Turner et al., 2009, 2014). Milder conditions will reduce establishment barriers for many native and non‐native species alike and may act to lengthen the growing season or activity window (Block & Convey, 2001; Convey, 2011; Hughes et al., 2006, 2013).

Antarctica's greatest barriers to alien species colonization, establishment and successful spread are its remoteness and extreme climatic conditions (Barnes et al., 2006; Bergstrom, 2022; Convey et al., 2006). A vast expanse of ocean and fierce winds act as a strong filter to natural dispersal of non‐native species to the continent, although natural dispersal events do occur (Barnes et al., 2006; Fraser et al., 2016, 2018). For example, marine organisms survive the crossing by hitchhiking on floating kelp, which readily traverses the Antarctic Polar Front (Avila et al., 2020; Fraser et al., 2016, 2018), and genetic and aerial modelling studies suggest that both *C. quitensis* and the moss *Chorisodontium aciphyllum* have dispersed multiple times from South America via animal (pearlwort) or wind (moss) vectors (Biersma et al., 2018, 2020). On top of natural dispersal opportunities, there are also growing opportunities for dispersal via human vectors (as noted above). These opportunities are likely to increase with expanding science and tourism activity on the continent, and indeed studies identifying sites at risk of non‐native species incursion flag human activity hotspots as being at high risk (Chown et al., 2012; Duffy & Lee, 2019; McCarthy et al., 2022; Pertierra, Hughes, et al., 2017), particularly those that are visited first by intercontinental ships (Bender et al., 2016).

Asides from its remoteness, Antarctica's other defence against non‐native species invasion is the climatic extremes: low temperatures, low water availability, high winds, freeze–thaw dynamics, and a short growing season (Bergstrom, 2022; Convey, 1996). Terrestrial Antarctic species often have key adaptations to cope with the extreme environment (Convey, 1996; Convey et al., 2014; Kennedy, 1999). Tardigrades and nematodes, for example, can undergo anhydrobiosis, completely desiccating until more suitable conditions occur (Adhikari et al., 2010; Tsujimoto et al., 2016), and similarly, bryophytes also desiccate and enter a suspended metabolic state until conditions improve (Bramley‐Alves et al., 2014). This explains why Antarctic vegetation is dominated by non‐vascular lichens and mosses, which are well equipped to survive the exceedingly dry, cold and nutrient‐poor conditions (Bramley‐Alves et al., 2014; Convey, 1996). Thus, even if a non‐native species dispersed, or was transported, to the white continent they often lack the capacity to survive its harsh environment (Barnes et al., 2006; Bergstrom et al., 2006; Hughes et al., 2006). However, as climate conditions become milder, this barrier will weaken and species previously unable to establish may now be able to take hold (Barnes et al., 2006; Bergstrom, 2022; Duffy et al., 2017; Frenot et al., 2005; Holland et al., 2021). This reality is reflected in both current observations of non‐native species spread and in projections for the future.

Over 15 non‐native invertebrate species have been recorded in the Antarctic Peninsula, with alien collembolas found in at least 26 locations (Hughes et al., 2015). A persistent invader is the flightless midge *Eretmoptera murphyi*, which is native to wetter, warmer sub‐Antarctic South Georgia and is believed to have been accidentally introduced to Signy Island in the 1960s. It has since spread over 85,000 m^2^ of the island at an accelerating rate (Bartlett et al., 2020). The midge is estimated to increase litter turnover by over nine times the rate of the native Signy soil community, indicating potential for substantial impacts on the ecosystem (Hughes et al., 2013). Furthermore, *E. murphyi* appears better equipped to utilize suitable activity windows than Antarctica's only native midge, *Belgica antarctica*. The invasive midge reproduces asexually rather than sexually (as native *B. antarctica* does) and may threaten the native species if introduced to the latter's range (Bartlett et al., 2019, 2020). To date, in continental Antarctica, non‐native invertebrates have only been recorded to survive within buildings and infrastructure associated with research stations. Most have been eradicated, although a fungus gnat population persists within Casey station (Bergstrom, Sharman, et al., 2018; Hughes et al., 2005, 2015).

For plants—there have been two records of removal in continental Antarctica, *Poa trivialis* at Syowa Station and several species together at Progress II Station, though there are currently no known established species in the environment (Hughes et al., 2015). In the Antarctic Peninsula region, a single clump of *P. pratensis* persisted for 60 years before its removal in 2015 (Pertierra, Aragón, et al., 2017) and there have been repeated occurrences (and removal) of the invasive grass *P. annua* (Chwedorzewska et al., 2014; Malfasi et al., 2020; Molina‐Montenegro et al., 2012, 2019). This species is substantially established and spreading on King George Island, including in the forefield moraines of retreating Ecology Glacier; with an eradication attempt ongoing (Galera et al., 2017, 2021; Olech & Chwedorzewska, 2011). Experimental studies found that *P. annua* was associated with a decrease in the biomass of both native vascular plant species and a reduction in their photosynthetic performance (Molina‐Montenegro et al., 2012). These results suggest that *P. annua* is likely to compete with, and may even outcompete, *D. antarctica* and *C. quitensis* if it were to become established at a broad scale, and this potential may worsen in a warmer and wetter future (Cavieres et al., 2018; Molina‐Montenegro et al., 2019).

Species distribution models predict broad swathes of the western Antarctic Peninsula are already climatically suitable for further range expansions of *P. annua*, *P. pratensis*, *E. murphyi* and the collembola *Hypogastrua viatica*, with sites of high human activity at greater risk (Bartlett et al., 2020; Pertierra et al., 2020; Pertierra, Hughes, et al., 2017; Vega et al., 2021). Indeed, they predict that 80% of the newly emerged ice‐free area (and >25% of all ice‐free area) will be vulnerable to invasion by one or more cold tolerant non‐native species by 2100 (Duffy & Lee, 2019). Germination experiments on non‐native plant species suggest the species distribution models may even underestimate the suitable range for invaders (Bokhorst et al., 2021).

It is not only non‐native species that will benefit from milder conditions, but also some native Antarctic species. As indicated above, growth and reproduction for terrestrial species are confined to small temporal windows in the Antarctic summer when conditions are milder, with adequate water and sunlight, to allow life to succeed (Bramley‐Alves et al., 2014; Convey, 1996). As temperatures warm and if water becomes more available, these activity windows will widen, potentially allowing species to become active earlier in the season and to remain active for longer (Block & Convey, 2001). There is evidence some species are already benefiting from warming temperatures. Antarctica's two vascular plants have expanded their ranges and abundance substantially since the 1960s, likely in response to increased summer temperatures (Cannone et al., 2016, 2022; Smith, 1994). Sampling across the maritime Antarctic has revealed that soil fungi increase in diversity with temperature, which could lead to increases in soil productivity as the region warms (Newsham et al., 2016). Bank‐forming mosses in the Antarctic Peninsula have also increased their growth rates, along with associated increases in microbial productivity, in response to warming post‐1950 (Amesbury et al., 2017). Increased temperatures in the shoulder periods of the main growing season were identified as particularly important (Amesbury et al., 2017). As bank‐forming mosses appear to be highly sensitive to temperature variations, large‐scale changes in distribution and growth of mosses and associated microbes are expected with climate change. Indeed, regional greening may already be underway (Amesbury et al., 2017). This contrasts with the drying trend and Antarctic ‘browning’ in East Antarctica—resultant reductions in hydric species and increases in xeric species abundances have been observed in the Windmill Islands (Robinson et al., 2018), while an extension of this pattern is the widespread distribution of moribund mosses reported for the Vestfold Hills (Bergstrom et al., 2021). Greening and browning trends are far more apparent across the Arctic and exhibit considerable regional variation (Ju & Masek, 2016; Phoenix & Treharne, 2022). Such varied responses across the Antarctic continent, and polar regions more generally, illustrate the complexities of understanding the responses of native biota to climate changes (Phoenix & Treharne, 2022).

For vertebrates, fur seals have been extending further south in recent decades, though it is currently unknown whether they are taking advantage of regional warming or are simply reclaiming their historical distribution post sealing (Smith, 1988). Whilst Adélie populations are declining in the Peninsula (Lynch et al., 2012; Santos et al., 2018), gentoo penguins are expanding their ranges southward and are colonizing recently deglaciated areas within their historic range (Herman et al., 2020). Predicted increases in coastal accessibility from reduced sea ice extent and the increasing availability of ice‐free area for breeding will undoubtedly benefit the gentoo penguin (Herman et al., 2020), even as similar changes elsewhere on the continent threaten species like the emperor penguin (Jenouvrier et al., 2021).

Invertebrate populations will also have variable responses to a milder climate. Earlier and increased water availability might benefit the collembola *Cryptopygus antarcticus*, but summer drought once meltwater supplies are exhausted may then act to restrict it (Block & Convey, 2001). Some soil arthropods in the maritime Antarctic, including *C. antarcticus*, may experience a slow decline with increasing temperatures (Bokhorst et al., 2008). Laboratory microcosm experiments on McMurdo Dry Valley soil communities demonstrated that while increasing frequency of freeze–thaw cycles reduced abundance across the board, warming led to increases in abundance of nematodes, while bacterial numbers decreased—suggesting a decoupling of predator–prey responses with warming (Knox et al., 2017). Similarly, long‐term observational studies from Taylor Valley find a variable response of soil microfauna to warming and increased soil moisture, with the dominant *S. lindsayae* declining, but increases in some rotifers, tardigrades, and other nematodes (Andriuzzi et al., 2018; Gooseff et al., 2017).

Spatially heterogeneous climatic changes will produce varying responses among populations in different regions of Antarctica, even within species of the same taxon. Understanding spatially and taxonomically variable responses of terrestrial biodiversity is important for future conservation efforts, where different management actions may be required in different regions. What exactly is it that makes some Antarctic species climate change winners and some losers? Sadly, true Antarctic specialists may be the ones to suffer the most. Emperor and Adélie penguins, the endemic moss *S. antarctici* and the dry‐tolerant nematode *S. lindsayae* thrive in Antarctica's extreme conditions, but with a changing climate and ever‐increasing human activity they may be at risk of inevitable declines (Andriuzzi et al., 2018; Cimino et al., 2016; Iles et al., 2020; Jenouvrier et al., 2020). Those species best able to capitalize on newly suitable conditions are likely those with inherently broad climatic tolerances or those expanding from more temperate areas. Many Antarctic microbes and plants grow optimally at higher temperatures (>15°C), but are capable of growing at much lower temperatures, allowing them to extend their activity window and maximize reproductive potential (Hughes et al., 2006; Perera‐Castro et al., 2020). And, as noted above, a number of species are already increasing abundance and/or distributions in response to warming. There remains some debate whether the life history of Antarctic species is characterized by flexibility, as might be expected if they invest in flexible strategies rather than reproduction (Convey et al., 2006; Turner et al., 2009), or whether they should be considered more rigid in their life histories compared with non‐native analogues because the maintenance of flexibility is energy intensive. For this reason, we do not have a general understanding of native species' capacity for successfully competing against non‐native colonizers.

## 4 HABITAT TRANSFORMATION BENEFITS SOME AND IS DETRIMENTAL TO OTHERS: BIODIVERSITY STARTS TO HOMOGENIZE ACROSS THE CONTINENT

The environmental changes discussed above will be beneficial to some species and harmful for others. It is likely that species interactions will change, and a system primarily built on abiotic drivers of distributions may start to shift towards a more biotically driven system (Convey, 2010; Nielsen & Wall, 2013). Increased competition for space and resources may lead to a loss of local endemism and the emergence of more similar communities across regions (as is being observed in urban areas globally; Concepción et al., 2015; Clergeau et al., 2006).

The role of biotic interactions in structuring the terrestrial communities of Antarctica is poorly understood and believed to be overshadowed by abiotic drivers such as water and nutrient availability (Convey et al., 2014; Hogg et al., 2006). Undoubtedly vegetation changes will drive changes in invertebrate responses, and there is some evidence that biotic factors will start to increase in importance with climate change. For example, abundance and diversity of microbial and invertebrate communities in the Antarctic Peninsula was lower in bare soils than in vegetated soils, with further variations based on climate severity and vegetation cover type (algae, lichens, moss, or grass; Ball et al., 2022). The current and predicted increases in vegetation with warming are expected to drive an associated shift in soil communities, and therefore, ecosystem function (Ball et al., 2022). In the Arctic, thawing permafrost has led to increasing microbial decomposition of soil organic matter, increasing available nutrients (C and N) and thus increasing vegetation productivity and changing community composition, and ultimately impacting the global carbon cycle (Heijmans et al., 2022; Keuper et al., 2012; Ricketts et al., 2020; Salmon et al., 2016). Similar effects may also be expected in Antarctica. For direct biotic interactions—in microcosm experiments of Dry Valley soils, *S. lindsayae* exerts top‐down control over its bacterial prey, yet environmental stress, particularly increased salinity, substantially altered the biotic interaction resulting in bacterial abundance increasing as *S. lindsayae* declined (Shaw & Wall, 2019). Decoupling in biotic interactions could have substantial impacts on terrestrial ecosystems. These studies highlight likely future changes in biotic interactions of *current* communities, but in addition to these are the introduced interactions from *different* communities potentially coming together in novel environments. A primary factor, particularly in relation to non‐natives species establishment, is competition.

Though there have been some studies examining competition between native and non‐native species, particularly with regards to the vascular plants (see above: Cavieres et al., 2018; Molina‐Montenegro et al., 2019, 2012), we know very little about how biotic interactions are likely to shape terrestrial biodiversity in the future. We need further research to better understand the competitive interactions among native taxa and between native taxa and their non‐native counterparts, and the flow‐on effects of these interactions on ecosystems. It is theorized that through investment in flexible life histories and stress tolerance adaptations that Antarctic species are generally poor competitors (Convey, 1996; Hughes et al., 2019). Examples from the sub‐Antarctic islands indicate that non‐native species in the region can be extremely competitive and have devastating, broadscale impacts on an entire ecosystem (Angel et al., 2009; Lebouvier et al., 2020; McGeoch et al., 2015). Does this mean that some native Antarctic species will start to be encroached on by more competitive species, or that communities will become more homogeneous? This prospect is certainly a common concern (Hughes et al., 2019; Lee et al., 2017), though we currently lack sufficient knowledge to confidently identify likely outcomes.

Many populations in terrestrial Antarctica have remained isolated from outside incursions for extended time periods, sometimes even millions of years (Convey et al., 2009; Convey & Stevens, 2007). These long separations have resulted in distinct communities, with some species, as far as we can tell based on survey data, endemic to even single ice‐free areas. For example, the rotifer *Rhinoglena kutikovae* is known only from the Bunger Hills, East Antarctica (De Smet & Gibson, 2008), and nunataks in inland Ellsworth Land are distinctive for having no nematodes (Convey & Mcinnes, 2005). Even within species, populations have sometimes accumulated high degrees of genetic diversity when separated from each other over even relatively short distances, for example, the highly divergent springtail populations in Victoria Land (Collins et al., 2019). Mixing of these populations, either through natural dispersal facilitated by habitat changes, or via assisted dispersal, could lead to a loss of genetic diversity within species. Indeed, a study on *G. hodgsoni* in Taylor Valley suggests that, with warming temperatures, one haplotype lineage could have a selective advantage over a more cold‐adapted lineage, potentially resulting in reduced genetic variability (Collins & Hogg, 2016). In contrast, increases in sexual reproduction, rather than asexual vegetative reproduction, could increase the genetic diversity of mosses—resulting also in greater dispersal, given that spores travel further than vegetative propagules (Casanova‐Katny et al., 2016; Smith & Convey, 2002). Ice melt or retreating glaciers may even expose currently entombed microbes, bryophytes, or even soil microfauna capable of resuming active states or regenerating after long time periods (Roads et al., 2014), as has been observed in Arctic ecosystems (La Farge et al., 2013; Miner et al., 2021; Shmakova et al., 2021). This could lead to further changes in community composition, biotic interactions, or ecosystem processes (Miner et al., 2021). As with the other hypotheses, it appears that there will likely be large amounts of taxonomic and spatial variability in response to climate change. Increasing levels of homogenization may occur in some places, especially if non‐native species outcompete their native counterparts (as studies on *P. annua* and *E. murphyi* suggest; Bartlett et al., 2019; Cavieres et al., 2018; Molina‐Montenegro et al., 2019).

Climate projections suggest it is unlikely there will be a broad scale trend towards biotic homogenization across the continent though, at least not in this century. Projections of changes in ice‐free area extent, and associated temperature and precipitation changes, suggest that impacts will be concentrated in the Antarctic Peninsula (Lee et al., 2017), with some increases also projected for other coastal sites (e.g. the Windmill Islands in East Antarctica; Robinson et al., 2018). These projections imply that, at least for the next 80 years, we might not see large climate‐associated shifts in the terrestrial communities of continental Antarctica, though this is likely to change as warming effects will continue well beyond the end of the century even with strong mitigation measures (IPCC, 2021). Importantly, current long‐term climate projections do not incorporate the impacts of extreme events, which will increase in frequency with warming (IPCC, 2021). Extreme events, such as the Dry Valley floods, can leave decadal signatures on populations, and unprecedented weather events, such as heatwaves, sea ice rafting, and transfer of moisture deep into the continent, can have regional to continental impacts (Bergstrom, Woehler, et al., 2018; Robinson et al., 2020).

**FIGURE 2:**
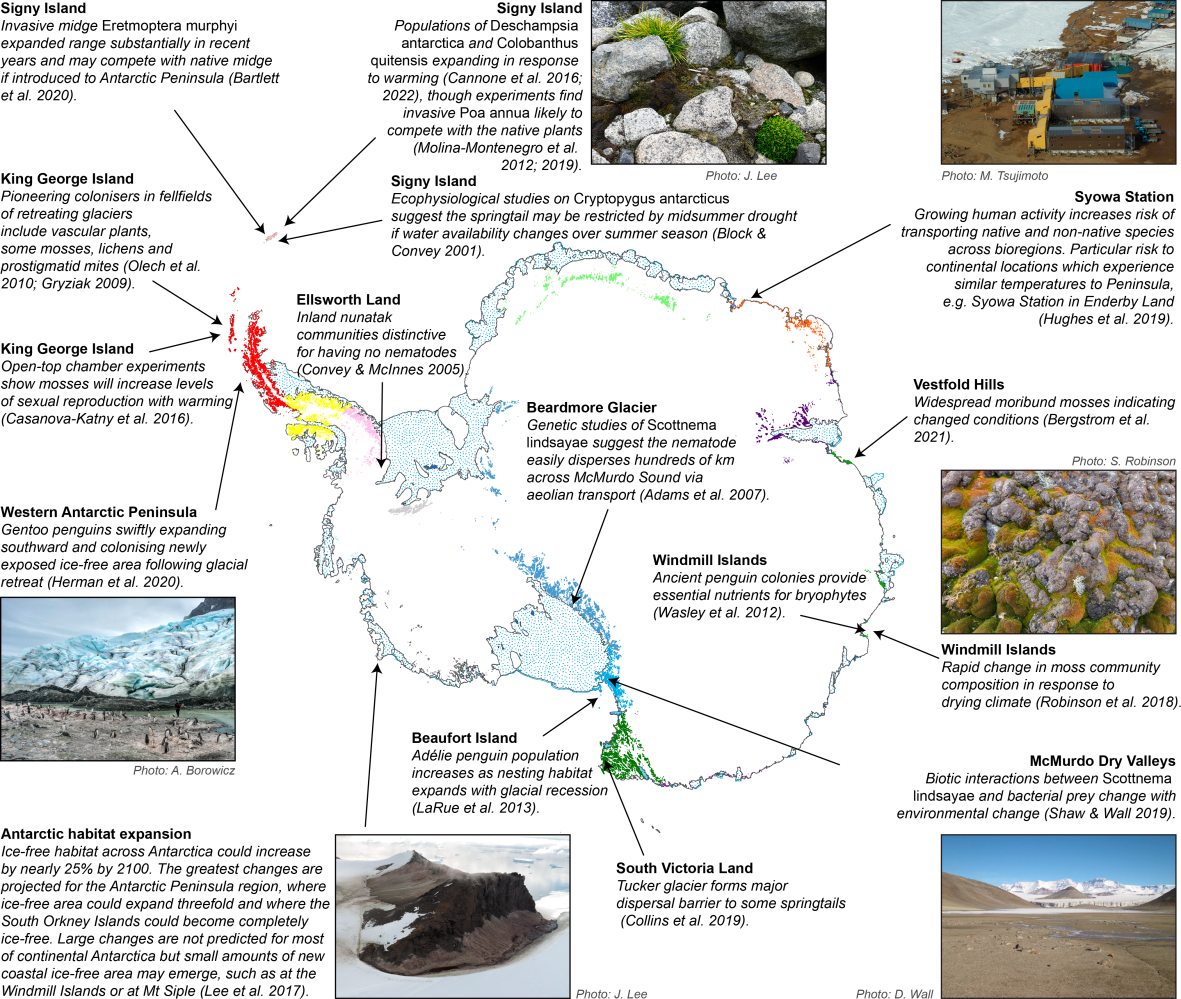
Various climate change impacts on terrestrial biodiversity across Antarctica and how future habitat transformation may affect them. Coloured patches represent ice‐free areas, where colour denotes different biogeographical regions (Terauds & Lee, 2016).

On‐ground observations show communities are already being impacted by climate change across the continent (see Figure 2; Andriuzzi et al., 2018; Cannone et al., 2022; Robinson et al., 2018), suggesting more subtle changes may be occurring at the microclimate scale than those reflected in broadscale models. Similar effects were found from a LTER research site in Low Arctic Alaska, where despite no significant trend in air temperatures apparent in the past 25 years, biochemical indicators revealed the ecosystem is responding to climate changes (Hobbie et al., 2017). In any case, changes in Antarctica may be expediated by growing human activity across the continent. Release of black carbon around research stations and popular tourist landing sites will accelerate snow and ice melt (Cordero et al., 2022), potentially expediating the expansion of new ice‐free areas around sites of human activity. Human movement between distinct sites, or bioregions, increases the risk of transporting both native and non‐native species to new destinations, facilitating potential competition or homogenization (Hughes et al., 2010, 2019; Vega et al., 2019). There could be substantial impacts if outsiders were inadvertently introduced to isolated or unique communities, for example, the introduction of nematodes to the inland nunataks of Ellsworth Land.

## 5 CONCLUSIONS

Through exploring several hypotheses regarding the future of Antarctica's ice‐free areas, we highlight that considerable uncertainty remains regarding the forecasted impacts of habitat transformation on terrestrial Antarctica. In part, this is due to considerable spatial, temporal, and taxonomic variation in biological responses to climate warming and associated expansion of ice‐free areas. This variation occurs even among species of the same taxonomic group (e.g. mosses and collembola), or between populations of the same species in different regions. In the short term, it seems clear that some native species are likely to benefit from climate change, yet it is unknown whether these benefits accumulate with further warming, or if there is a threshold beyond which current climate change ‘winners’ start to decline. Changes in biotic interactions among species, such as a decoupling of consumers and their food sources, may also trigger future declines. It is also evident that we know a lot about some species, such as *D. antarctica*, *S. lindsayae* and *C. antarcticus*, yet we know very little about some of their closest Antarctic relatives. Better understanding variation in species responses will contribute crucial information for future conservation planning and management decisions. Pre‐emptive management actions are likely to be far more successful than implementing actions in the future for populations already in decline. Bergstrom et al. (2021) give some examples of pre‐emptive management such as, hydro‐ecological engineering to replace lost water (e.g. building of strategically placed snow fences), translocation of biota to areas with more secure future water availability, or actively creating new habitats. Certainly, it is better to prevent the introduction of non‐native species (or facilitating native species dispersal to new sites) than attempt to eradicate or manage them once established (consider Galera et al., 2017). Yet, we also need to engage decision‐makers and managers in discussions around management of non‐native, or native, species that arrive via natural dispersal—particularly those that may not have established without the assistance of anthropogenic climate change. Should these species be eradicated or managed, or should they be counted as new natives (see Bergstrom, 2022 for decision tree on management options)? What can we do for species that are already declining? Are we trying to conserve the state of Antarctic ecosystems benchmarked to some historic past state, or are we interested in conserving ecosystem processes, even when some of those processes may result in the elimination of some endemic species? Ultimately, we do not yet fully understand what direct and indirect impacts of climate change mean for terrestrial biodiversity, and whether we can mitigate some of these changes, or even if we wish to maintain the status quo. We urgently need further research to address these gaps (Box 1 and Table S1).

Box 1 **Ten key research questions to be addressed in future interdisciplinary research efforts to further understanding of the impacts of habitat transformation on terrestrial Antarctic biodiversity and to test the broad hypotheses proposed in this paper. See Table S1 for a more extensive list of potential research questions to help address knowledge gaps.**

Key future research questions

1. How will ice‐free area expansion drive and interact with changes in water availability, how will this vary between regions or patches of different shape and size, and what impacts will this have on biodiversity?
2. Will newly exposed ice‐free areas host suitable soil properties needed for biodiversity establishment, and will these properties be the same across Antarctic environments and regions?
3. How far and how quickly do terrestrial species disperse and how does changing structural connectivity impact functional connectivity for different taxa?
4. Are colonization processes universal across space and will the structure of Antarctic communities be highly altered in the future?
5. Will those species that may benefit from climate change in the short term continue to benefit in the long term?
6. Will abiotic drivers of species diversity and distribution shift towards more biotic drivers as climate change progresses, and will there be decoupling of biotic interactions between species?
7. Will a milder climate and increasing competition result in local homogenization of terrestrial communities, and how would a substantial loss of biodiversity impact ecosystem function?
8. Should we manage non‐native or native species if they have arrived through natural means?
9. How will human activities directly alter the ice‐free landscape and how will this interact with climate driven changes?
10. How do we best mitigate impacts on terrestrial biodiversity and what environmental state are we trying to preserve?

## AUTHORS' CONTRIBUTIONS

J. R. L, J. D. S. and S. A. R. conceptualized the project. All authors contributed to writing the manuscript.

## FUNDING INFORMATION

J. R. L. is supported by a Royal Commission for the Exhibition of 1851 Research Fellowship. D. H. W. acknowledges funding from NSF OPP 1341648, NSF OPP 1115245. This work was supported by ARC SRIEAS Grant SR200100005 Securing Antarctica's Environmental Future (S. A. R., J. D. S.) and DP200100223 (S. A. R., M. W.)

## Supporting information


Table S1

## Data Availability

Data sharing not applicable to this article as no datasets were generated or analyzed during the current study.
